# Control of Tumors by Antigen-Specific CD8^+^ T Cells through PDL1-Targeted Delivery of Antigenic Peptide

**DOI:** 10.1155/2022/9054569

**Published:** 2022-01-04

**Authors:** Po-Hao Feng, Xiaoxu Wang, Louise Ferrall, T.-C. Wu, Chien-Fu Hung

**Affiliations:** ^1^Division of Pulmonary Medicine, Department of Internal Medicine, School of Medicine, Taipei Medical University, Taipei, Taiwan; ^2^Department of Pathology, Immunology Graduate Program, The Johns Hopkins University, Baltimore, MD, USA; ^3^Department of Pathology, The Johns Hopkins University, Baltimore, MD, USA; ^4^The Johns Hopkins University, Oncology, Baltimore, MD, USA; ^5^The Johns Hopkins University, Obstetrics and Gynecology, Baltimore, MD, USA; ^6^The Johns Hopkins University, Molecular Microbiology and Immunology, Baltimore, MD, USA

## Abstract

Tumor antigen-specific T cell function is limited by immune tolerance in the tumor microenvironment. In the tumor microenvironment, tumor cells upregulate PD-L1 expression to promote T cell exhaustion by PD-1/PD-L1 interactions and undergo mutations to avoid being targeted by tumor antigen-specific T cells. Thus, tumor cells escape the immune surveillance by causing immune tolerance. We reason that a chimeric molecule made of a PD-L1-specific antibody linked to a cleavable antigenic peptide can target the antigenic peptide to the tumor microenvironment, resulting in the blockade of the PD-1/PD-L1 pathway and killing tumor cells through the coating of antigenic peptide. Here, we have generated a therapeutic chimeric protein containing the PD-L1 single-chain variable fragment (scFv) linked to a cleavable model cytotoxic T lymphocyte (CTL) epitope: E7 CTL peptide. Our study demonstrated that our chimeric protein (named PDL1-scFv-Fc-RE7) can target PD-L1-expressing tumor cells and enable E7 presentation by releasing cleavable E7 CTL peptide to coat tumor cells, resulting in tumor clearance by E7-specific CD8^+^ T cells. The presentation of the E7 peptide by cancer cells can then render tumor cells susceptible to the killing of preexisting E7-specific CD8^+^ T cells and contribute to tumor clearance. Our finding suggests a synergistic approach to not only enhance antigen-specific tumor clearance but also bypass immune tolerance.

## 1. Introduction

The ability of tumor cells to downregulate the immune system and avoid recognition by cytotoxic T cells is one of the major barriers for CD8^+^ T cell-mediated tumor clearance. Mechanisms that result in tumor evasion and immune downregulation include immune suppression and immunoediting [1, 2]. The most well-established immune suppression mechanism relies on the PD-1/PD-L1 axis. PD-L1 (B7-H1; CD274) belongs to the B7 superfamily and is one of the ligands of PD-1, which is expressed on T cells. Upon PD-1/PD-L1 binding, PD-1 recruits SHP2 phosphatase, which dephosphorylates the CD3*ζ* ITAM sites of T cell receptors (TCR) and sites on CD28, thereby attenuating TCR/CD28 signaling and impeding T cell activation [3, 4]. In order to suppress the immune system, tumor cells often express surface PD-L1 [5]. Since PD-L1 expression can be induced by IFN-*γ* from lymphocytes, PD-L1 is an important molecule that facilitates tumor escape by suppressing lymphocyte function [6, 7]. Due to the important immune suppressive function of PD-L1, many antibody drugs that block the interaction between PD-1 and PD-L1, such as Atezolizumab, Durvalumab, and Avelumab, have been approved by the FDA [8].

Another mechanism of tumor immune evasion is immunoediting (for review see [1]). With the pressure of immunoselection, the genetically unstable initial tumor cells finally evolve into tumor variants that escape the immune surveillance [1]. Cancer immunoediting enables tumor cells to avoid antigen-specific T cells by not expressing surface antigen recognizable by activated cytotoxic T cells and thus represents an obstacle for the application of antigen-specific cancer immunotherapy. In order to reverse immunoediting, we have previously reported on a strategy to coat tumor cells with foreign antigen that is recognized by preexisting cytotoxic T cells to generate antitumor effects [9]. Once the tumor is coated with and presenting the foreign antigen via the major histocompatibility complex (MHC-I), preexisting antigen-specific CD8^+^ T cells will be recruited to target the tumor [9].

However, even if tumor-specific antigens were expressed, tumor cells could still evade clearance by the immune system if there is heightened immunosuppression due to the PD-L1/PD-1 interaction. Therefore, in order to most effectively clear tumor, immunosuppression must be reversed, and tumor cells must be made recognizable by cytotoxic T cells. To overcome these two barriers for tumor clearance, we designed a universal therapy that targets PD-L1 overexpressing tumor cells and coats them with foreign antigens to induce a stronger antigen-specific antitumor immune response.

Immunotherapeutic T cell vaccines function by priming T cells to target a particular antigen presented by MHC-I on target cells. DNA vaccines are especially promising forms of immunotherapeutic vaccine due to their simplicity and high safety profiles. pcDNA3-CRTE7 DNA vaccine is a therapeutic vaccine that has previously been shown to activate CD8^+^ T cells to target human papillomavirus type 16 (HPV16) E7 antigen [10–12]. The pcDNA3 plasmid is encoded with calreticulin (CRT), which enhances major histocompatibility complex class I (MHC-I) antigen presentation of HPV16 E7 and has an antiangiogenic effect. The vaccine has demonstrated an antitumor effect against HPV16 E7-expressing tumors and generates appreciable HPV16 E7-specific CD8^+^ T cell responses [10].

In this study, we hypothesized that the modified PD-L1 single-chain antibody (scFv) fused with foreign peptides can not only inhibit PD-L1-mediated T cell suppression but also elicit foreign peptide-specific T cell-mediated antitumor effects without immunoediting of tumor antigens. To test our hypothesis, we generated a chimeric protein that has a tumor-homing module and a functional module. The tumor-homing module is the PDL1-scFv cloned from the fully humanized PDL1 antibody Atezolizumab. PDL1-scFv has good cross-reactivity between humans and mice. The functional domain includes human IgG1 Fc region and a foreign peptide E7 peptide from HPV16 that flanked by a furin cleavage site (R). If tumor cells express furin, the HPV E7 peptide will be cleaved from the PDL1-scFv antibody portion and subsequently loaded into MHC-I, because furin can cleave the amino acid sequence RVKR specifically. This will hypothetically result in a tumor cell coated with foreign antigens.

Here, we found that the chimeric protein PDL1-scFv-Fc-RE7 can be efficiently expressed by transfected cells. In addition, we showed that cells expressing PDL1 can be bound by the chimeric PDL1-scFv-Fc-RE7 proteins. Furthermore, we demonstrated that the binding of the chimeric PDL1-scFv-Fc-RE7 protein by PD-L1-expressing tumor cell led to the release of E7-specific CTL epitope and the presentation of the E7 CTL epitope by the MHC-I molecules of tumor cells, rendering them susceptible to E7-specific CTL-mediated killing by preexisting E7-specific CD8^+^ T cells generated by CRTE7 DNA vaccine. More importantly, we observed that E7 vaccine-vaccinated tumor-bearing mice treated with the chimeric PDL1-scFv-Fc-RE7 protein were able to lead to the control of tumor in vivo. Our data serve as important foundation for future clinical translation.

## 2. Materials and Methods

### 2.1. Plasmid DNA Construction and Preparation

We utilized a cloning strategy that was described previously [13]. In short, the Atezolizumab PDL1-scFv sequence was obtained from the publication of Lee et al. [8] and synthesized by GeneScript (Piscataway, NJ). PDL1-scFv was cloned into EcoRI/Bgl II sites of pFUSE-hIgG1-Fc2 purchased from InvivoGen (San Diego, CA). To generate PDL1-scFv-Fc-RE7, the DNA fragment encoding the DNA furin cleavage site (RVKR) linked to the E7 peptide (amino acid 49-57, RAHYNIVTF) was generated using oligos CTAGAAGGGTGAAGAGACGCGCTCACTACAACATCGTGACCTTTTAAC and TCGAGTTAAAAGGTCACGATGTTGTAGTGAGCGCGTCTCTTCACCCTT, which were then annealed and cloned into XbalI/Xho sites of PDL1-scFv-Fc.

### 2.2. Transfection and Protein Purification

To get purified chimeric protein, 50 *μ*g of PDL1-scFv-Fc-RE7 and PDL1-scFv-Fc constructs was transfected into Expi293F cells (Gibco; Thermo Fisher Scientific, Inc. USA) in T-150 flasks using the Expi293™ Expression System Kit (Catalog #: A14635. Gibco; Thermo Fisher Scientific, Inc. USA). ExpiFectamine™ 293 Transfection Enhancers 1 and 2 were added one day after transfection. Five days later, the supernatants of cell cultures were collected and filtered with a 0.22 *μ*m syringe filter (Millipore, Billerica, MA, USA). We then applied the filtered supernatant to a HiTrap Protein G HP column (GE Healthcare) to get purified chimeric protein. Protein dialysis was done overnight with PBS buffer. PBS buffer was changed more than three times during dialysis. The concentration of purified protein was determined with a Bio-Rad Protein Assay Dye (cat #: 5000006, Bio-Rad). Finally, the protein purity was tested through sodium dodecyl sulfate-polyacrylamide gel electrophoresis (SDS-PAGE).

### 2.3. Cell Staining and Flow Cytometry Analyses

To assess PD-L1 expressions on tumor cell lines, different tumor cell lines were cultured with or without IFN-*γ* (Schering Corporation, Bloomfield, NJ, USA) for 16 hrs. Tumor cells were then stained with either the APC anti-mouse PD-L1 antibody (Clone 10F.9G2, BioLegend) or APC Rat IgG2b, *κ* isotype control (RTK4530, BioLegend). For chimeric antibody binding affinity assessments, 1 *μ*g of PDL1-scFv-Fc and PDL1-scFv-Fc-RE7 was incubated with tumor cells separately for 1 hr. Anti-human IgG (Fc-specific)-PE (eBioscience) was used as the secondary antibody. The percentage of E7-specific CD8^+^ T cells was determined by intracellular IFN-*γ* staining and then analyzed by FACScan analysis with the CELLQuest software (Becton Dickinson Immunocytometry System, Mountain View, USA).

### 2.4. E7-Specific T Cell Activation and *In Vitro* Cytotoxicity Assays

To test T cell activation, 2 × 10^4^/well ID8-luc cells were seeded into 96-well flat-bottom plates and cultured overnight. Five *μ*g/mL of chimeric protein was subsequently added, and cells were incubated at 37°C for 1 hr. After washing away the unbinding protein, 2 × 10^5^/well E7-specific cytotoxic T cells (CTLs) treated with 1 *μ*L/mL of Golgi Plug ™ Protein Transport Inhibitor (BD Biosciences, San Jose, CA) were added into each well. After overnight activation, E7 CTLs were subsequently stained with rat anti-CD8a-PE (Clone 53-6.7, BD Biosciences, San Jose, CA) and intracellular anti-IFN-*γ* FITC antibody (Clone XMG1.2, BD Biosciences, San Jose, CA). Finally, samples were analyzed. Similarly, to test the *in vitro* cytotoxicity of E7-specific CTLs, 5 × 10^3^/well ID8-luc cells were added to 96-well flat-bottom plates and cultured overnight. On the next day, cells were incubated with various concentrations of the chimeric protein at 37°C for 1 hr. After removing the media, 0 cells/well, 5 × 10^3^/well, or 5 × 10^4^/well E7-specific CTLs were added to the wells. The degree of CTL-mediated killing of tumor cells was measured by the IVIS luminescence imaging system series 2000 as described previously [9].

### 2.5. In Vivo Tumor Experiment

C57BL/6 mice (5 per group) were vaccinated twice at one-week intervals with 10 *μ*g pcDNA3-CRTE7 DNA plasmid vaccine intramuscularly followed by electroporation, which has been described previously [10]. One week after the second vaccination, the mice were challenged with 2 × 10^5^ MC38 tumor cells as described previously. Six days following the tumor challenge, tumor-bearing mice were treated with intramuscular 30 *μ*g PDL1-scFv-Fc-R7, PDL1-scFv-Fc, or no treatment twice at 3-day intervals. Mice were monitored for signs of stress and tumor growth. The survival of tumor-bearing mice was followed. All mice were treated and housed in accordance with the Johns Hopkins Animal Care and Use Committee guidelines.

### 2.6. Statistical Analyses

The data presented in this study are representative of at least two experiments performed and are expressed as means ± standard deviation (SD). The number of samples in each group for any given experiment was more than 3. Comparisons between individual data points were performed using Student's *t*-test. Statistical analysis was performed with GraphPad Prism 6.0 software. Tumor-bearing mice treated with different regimens were analyzed with Kaplan–Meier survival analysis. *p* values are indicated as one star (∗) if *p* < 0.05, two stars (∗∗) if *p* < 0.01, three stars (∗∗∗) if *p* < 0.001, and four stars (∗∗∗∗) if *p* < 0.0001.

## 3. Results

### 3.1. Western Blot Analysis Demonstrates the Purity of the PDL1-scFv-Fc-RE7 and PDL1-scFv-Fc Chimeric Proteins

**Figure 1:**
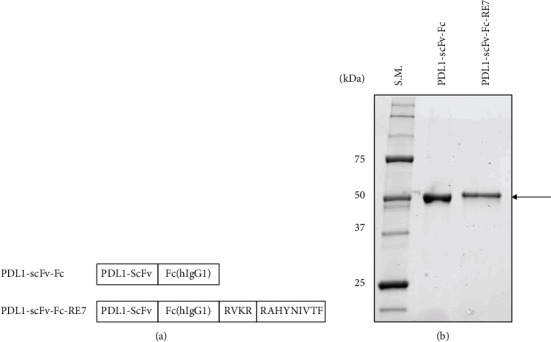
Generation and characterization of therapeutic chimeric proteins. (a) Schematic diagram of chimeric protein containing PDL1-scFv, Fc portion of human IgG1, furin cleavage site (RVKR), and E7 peptide (RAHYNIVTF). (b) SDS-PAGE of purified chimeric protein from Expi293F cells transfected with PD-L1-scFv-Fc and PD-L1-scFv-Fc-RE7 constructs. Arrow pointing at the bars indicating the size of PDL1-scFv-Fc and PDL1-scFv-Fc-RE7.

We generated constructs that encode chimeric proteins PDL1-scFv-Fc-RE7 and PDL1-scFv-Fc. The chimeric protein PDL1-scFv-Fc-RE7 contains Atezolizumab PDL1-specific single-chain variable region (PDL1-scFv) and hIgG1Fc region (Fc) linked to the E7 peptide by furin cleavage site (R). The chimeric protein PDL1-scFv-Fc is the same as PDL1-scFv-Fc-RE7 except without the furin cleavage site and E7 peptide. The schematic diagram of two chimeric protein constructs is shown in Figure 1(a). Next, we purified chimeric protein PDL1-scFv-Fc-RE7 and PDL1-scFv-Fc from the media supernatant of the transfected Expi293F cells (Gibco; Thermo Fisher Scientific, Inc. USA). The purified protein was demonstrated by SDS-PAGE (Figure 1(b)). The arrow indicates the position of the purified chimeric proteins that are highly pure. PDL1-scFv-Fc and PDL1-scFv-Fc-RE7 have similar sizes because the E7 peptide and furin cleavage site are only 13 amino acids long.

### 3.2. The Chimeric Proteins PDL1-scFv-Fc-RE7 and PDL1-scFv-Fc Successfully Bind to ID8-luc Tumor Cells

**Figure 2:**
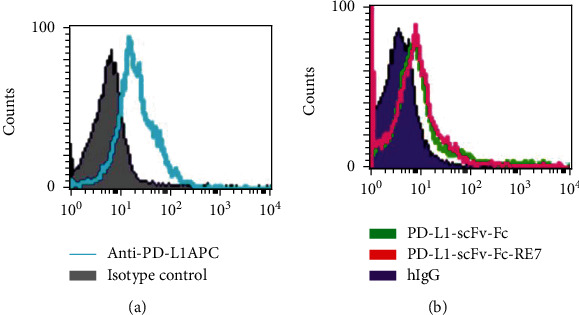
Characterization of the binding of PD-L1-scFv-Fc and PD-L1-scFv-Fc-RE7 to ID8-luc tumor cells using flow cytometry. (a) Representative flow cytometry analysis of commercial antibody bound to PD-L1 (blue clear histogram) or isotype control (shaded histogram). (b) Representative flow cytometry analysis of PDL1-SCFV (green), PDL1-SCFV-RE7 (red), or isotype control (purple shaded histogram).

We first tested PD-L1 expression level on murine ovarian tumor cell line ID8 expressing luciferin (ID8-luc) (Figure 2(a)) with a commercial anti-PD-L1 antibody. We selected the ID8 cell line because ID8 were demonstrated to have appreciable furin expression [9]. We then tested the ability of the PDL1-scFv-Fc-RE7 chimeric antibody to bind to the ID8-luc tumor cell lines. As shown in Figure 2(b), both PDL1-scFv-Fc-RE7 and PDL1-scFv-Fc chimeric proteins successfully bind to tumor cell with similar affinity to each other and the commercial anti-PD-L1 antibody. This demonstrates the specificity of the chimeric proteins to target the PD-L1-expressing tumors.

### 3.3. PDL1-scFv-Fc-RE7 Binds PDL1-Expressing Tumor Cells *In Vitro* and Leads to MHC Class I Presentation of the E7 Peptide to E7-Specific CD8^+^ T Cells

**Figure 3:**
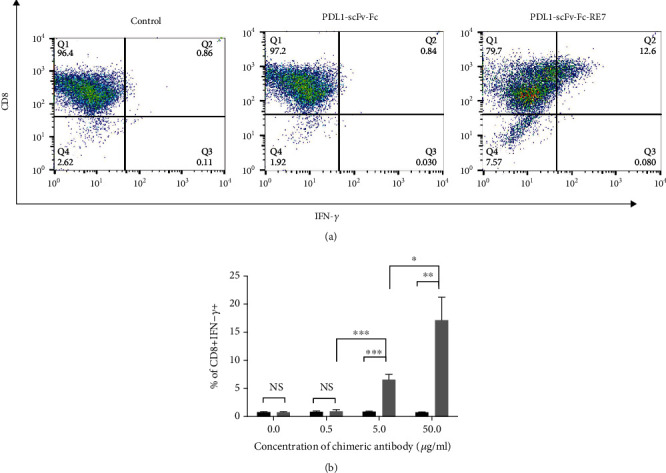
Activation of E7-specific CD8+ T cells by ID8-luc cells pretreated with PDL1-scFv-Fc or PDL1-scFc-Fc-RE7. (a) Representative flow cytometry analysis to demonstrate the activation of E7-specific CD8+ T cells following the incubation of ID8-luc cell pretreatment either 50 *μ*g/mL of PDL1-scFv-Fc or PDL1-scFc-Fc-RE7. ID8-luc cells without treatment are included as a negative control. E7-specific CD8+ T cell activation was determined by CD8 and IFN-*γ* staining. (b) Representative bar graph depicting the % of IFN-*γ*-secreting E7-specific CD8+ T cells out of total E7-specific T cells (mean ± SD). *p* values are indicated as one star (∗) if *p* < 0.05, two stars (∗∗) if *p* < 0.01, and three stars (∗∗∗) if *p* < 0.001.

We tested whether the binding of the PDL1-scFv-Fc-RE7 chimeric protein delivers the E7 peptide to MHC-I molecules to activate E7-specific CD8^+^ T cells. As shown in Figure 3(a), although PDL1-scFv-Fc shared similar binding affinity with PDL1-scFv-Fc-RE7 (Figure 2(b)), only PDL1-scFv-Fc-RE7 enabled E7-specific CD8^+^ T cell proliferation *in vitro*. When MHC-I cells present the E7 antigen that is cleaved off from the chimeric protein, E7-specific CD8^+^ T cells are activated and can target E7 antigen-coated tumor cells. PDL1-scFv-Fc did not similarly induce an E7-specific CD8^+^ T cell response. The percentages of CD8^+^ IFN-*γ*^+^ T cells were also assessed under various concentrations of chimeric antibody in triplicates (Figure 3(b)). Consistent with Figure 3(a), the percentages of CD8^+^ IFN-*γ*^+^ E7-specific T cells were significant when ID8-luc was incubated with PDL1-scFv-Fc-RE7 above 5 *μ*g/mL, whereas ID8-luc incubated with PDL1-scFv-Fc did not demonstrate high levels of CD8^+^ IFN-*γ*^+^ E7-specific T cells. This demonstrates that binding of PDL1-scFv-Fc-RE7 to PDL1-expressing tumor cells leads to the cleavage and subsequent release of the E7 antigenic peptide. The E7 peptides are then presented by MHC-I molecules of the tumor cells, resulting in the activation of E7-specific CD8^+^ T cells.

### 3.4. The Binding of PDL1-scFv-Fc-RE7 to PD-L1 Ligand-Expressing Tumor Cells Renders Tumor Cells Susceptible to E7-Specific CD8^+^ T Cell-Mediated Killing

**Figure 4:**
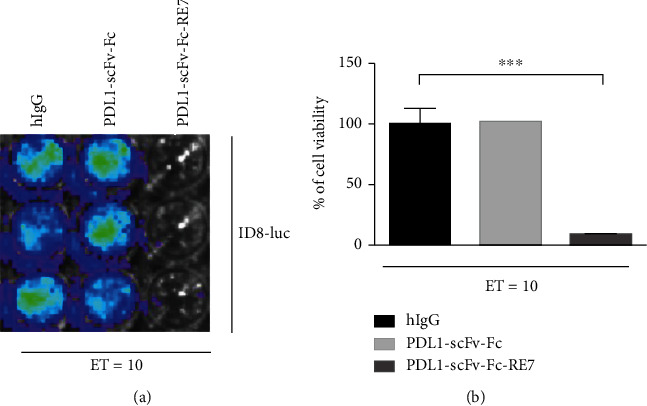
Characterization of the CTL-mediated killing of ID8-luc tumor cells incubated with the various chimeric proteins. (a) Representative luminescence imaging of in vitro E7-specific CTL killing of luciferase-expressing ID8-luc cells. ID8-luc cells were preincubated with 5 *μ*g/mL PD-L1-scFv-Fc, PD-L1-scFv-Fc-RE7, or hIgG. Pretreated cells were then incubated with E7-specific CD8+ T cells (*E*/*T* = 10/1). CTL-mediated tumor cell death was determined by decreasing luminescence activity. (b) Bar graph depiction of tumor cell viability after treatment with PD-L1-scFv-Fc, PD-L1-scFv-Fc-RE7, or hIgG protein (mean ± SD) (data representative of three experiments).

To determine if the binding of PDL1-scFv-Fc-RE7 with the ID8 luciferase-expressing tumor cells can render the tumor cell susceptible to E7-specific CD8^+^ T cell-mediated killing, the percentages of viable ID8-luc were measured by luciferase activity after incubating the cells with E7-specific CD8^+^ T cells. The viability of ID8-luc correlates with the luminescence signal measured. The presentation of E7 by the ID8-luc tumor cells will render the cells susceptible to E7-specific CD8^+^ T cell-mediated killing, resulting in diminished luminescence signal. As shown via luminescence imaging in Figure 4(a), the ID8-luc cells that were incubated with PDL1-scFv-Fc-RE7 had the lowest tumor cell viability (nondetectable luciferase activity). The ID8-luc cells incubated with PDL1-scFv-Fc were not susceptible to E7-specific CD8^+^ T cells due to the absence of E7 peptide presentation (detectable luciferase activity). No differences were observed in cells incubated with PDL1-scFv-Fc compared to cells that were incubated with hlgG isotype. The tumor cell viability in different conditions was summarized and analyzed in Figure 4(b). Thus, the binding of PDL1-scFv-Fc-RE7 has the ability to cause MHC-I presentation of E7 antigen by tumor cell, rendering the tumor cell susceptible to E7-specific CD8^+^ T cell-mediated killing.

### 3.5. Treatment of MC38 Tumor-Bearing Mice with PDL1-scFv-Fc-RE7 Leads to Potent Therapeutic Antitumor Effects

**Figure 5:**
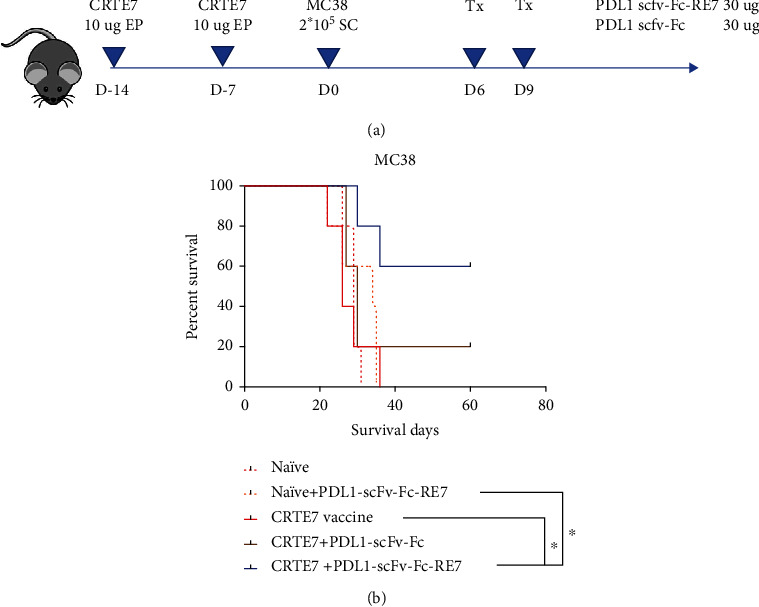
In vivo tumor treatment experiment using MC38 tumor-bearing mice treated with either PD-L1-scFv-Fc or PD-L1-scFv-Fc-RE7. (a) Schematic diagram of the experiment. C57BL/6 mice (5 per group) were vaccinated twice with 10 *μ*g pcDNA3-CRTE7 DNA vaccine intramuscularly followed by electroporation at one-week intervals. Vaccinated mice were challenged with 2 × 10^5^ MC38 tumor cells one-week postfinal vaccination. Tumor-injected mice were then treated with 30 *μ*g per mouse of PDL1-scfv-Fc-R7, PDL1-scfv-Fc, or no treatment twice at 3-day intervals starting 6 days after MC38 tumor challenge. (b) Kaplan–Meier survival was analyzed in tumor-bearing mice treated with different regimens.

To create a preexisting HPV16-16 E7-specific CD8^+^ T cell-mediated immune responses, we have used a potent therapeutic HPV DNA vaccine: CRTE7 DNA (for reference see [10]). C57BL/6 mice (5 per group) were vaccinated twice with 10 *μ*g pcDNA3-CRTE7 [10] intramuscularly followed by electroporation at one-week intervals. Mice were challenged with 2 × 10^5^ MC38 tumors one week after the last vaccination. Six days after tumor challenge, mice were treated with 30 *μ*g PDL1-scFv-Fc-R7, PDL1-scFv-Fc, or no treatment twice at 3-day intervals (Figure 5(a)). Kaplan–Meier survival was used to analyze tumor-bearing mice treated with different regimens. Mice treated with DNA vaccine pcDNA3-CRTE7 and PDL1-scFv-Fc-R7 had a significantly better survival at day 60 compared with other groups (Figure 5(b)). In comparison, mice who did not receive the CRTE7 DNA prior to treatment with PDL1-scFv-Fc-RE7 did not mount an appreciable therapeutic antitumor effect. Furthermore, mice that received CRTE7 vaccine alone also did not mount an appreciable therapeutic antitumor effect against MC38 tumor, which does not express HPVE7. Taken together, our results indicate that preexisting HPV16 E7 antigen-specific CD8^+^ T cells are crucial for the observed therapeutic effect mediated by PDL1-scFv-Fc-RE7 chimeric protein.

## 4. Discussion

We have successfully generated a novel chimeric protein PDL1-scFv-FC-RE7 for cancer immunotherapy. Our preliminary study has shown that linkage of a cleavable E7 CTL peptide to the PD-L1-specific single-chain variable region results in a chimeric molecule that may both reverse immune suppression via the PD-1/PD-L1 axis blockade and permit antigen-specific CD8^+^ T cell-mediated antitumor immunity. We found that PDL1-scFv-Fc-RE7 and PDL1-scFv-Fc both can bind tumor cells, as demonstrated by our ID8-luc murine ovarian tumor cell line model (Figure 2). As tumor expresses PDL1 on its surface, the PDL1-scFv region routes the chimeric molecule to the tumor site with significant specificity. This binding prevents T cells expressing PD-1 from binding PDL1 on tumor cells and thus prevents the T cell exhaustion and immune suppression while avoiding off-target effects. The addition of the E7-specific CTL peptide via the furin cleavage site resulted in MHC-I presentation of the E7 peptide in tumor cells exposed to PDL1-scFv-Fc-RE7 (see Figure 3). As the PDL1-scFv-Fc-RE7 protein binds tumor, cells that express furin will cleave the E7 protein and present it via MHC-I. Once presented, E7-specific CD8^+^ T cells can target the tumor, resulting in the apparent ability of PDL1-scFv-FC-RE7 to bypass immunoediting barriers and result in better tumor clearance than PDL1-scFv alone. Of note, there need to be preexisting cytotoxic T cell populations targeting the foreign antigen. If there is no preexisting E7-specific CD8^+^ T cell population, PDL1-scFv-Fc-RE7 cannot elicit similarly potent antitumor effects (Figure 5(b)).

In our study, we have observed the significance of the coating tumor with foreign antigenic peptide as an innovative strategy to take advantage of preexisting immunity for the control of tumor. ID8-luc cells that were treated with PDL1-scFv-Fc-RE7 were more readily targeted by E7-specific CD8^+^ T cell-mediated killing than ID8-luc cells treated with PDL1-scFv, demonstrating the additional antitumor immunogenicity resultant from the release of antigenic peptide from the chimeric protein (see Figure 4). The activation of E7-specific CD8^+^ T cell levels by the chimeric proteins was dose dependent on the concentration of PDL1-scFv-Fc-RE7, only significantly increasing when cells were incubated with PDL1-scFv-Fc-RE7 above 5 *μ*g/mL (Figure 3(b)).

Our studies serve as an important foundation for the approach of binding a tumor antigen to an anti-PDL1 agent to create a chimeric therapeutic protein. This technology holds significant promise and translational potential. We have previously created similar treatment for cancer based upon the NKG2D-ovarian cancer honing molecule [14, 15] or the Annexin A5 molecule [16, 17], which showed the potential clinical applications and the importance of using antigen-specific immunotherapy to reverse immune tolerance. Additionally, we have demonstrated that coating the tumor with antigenic peptide, as is done here with E7, makes tumor cells susceptible to CD8^+^ T cell-mediated killing [9]. Therefore, this new technology has potential to target multiple tumor types, as PDL1-scFv is not tumor specific, and coat the tumor cell with an antigen that will recruit preexisting antigen-specific CD8^+^ T cells to target the tumor.

One of the major limitations of the current study is that it requires the expression of PDL1 by the tumor cells. Not all tumors have been shown to highly express PDL1 [5, 18]. However, it has been demonstrated that cytokines, such as IFN-*γ*, are able to induce the expression of PDL1 by tumor cells [19]. It is conceivable that our current approach could potentially be enhanced by the combination of therapy with IFN-*γ* [20]. It would be of future interest to test such an approach in a relevant preclinical model. Such information would be critical for further clinical translation.

Our approach can be potentially extended to multiple different types of tumors in the body. This is because the tumor does not need to express E7 prior to treatment. As long as the chimeric protein is able to hone to the tumor, it will be able to coat the tumor with E7 antigenic peptide, rendering them susceptible to the E7 antigen-specific CD8^+^ T cell-mediated killing. Currently, there are multiple therapeutic HPV vaccines targeting E7 in clinical development (for review [21]). These therapeutic HPV vaccines potentially can be used to create an HPV16 E7 antigen-specific CD8^+^ T cell response prior to the administration of PDL1-scFv-Fc-RE7.

The current approach can also be employed using foreign antigens other than HPV16 E7. PDL1-scFv-Fc can be linked to an alternate peptide for which the patient already has existing CD8^+^ T cell immunity. For instance, the protein can include cleavable foreign antigen peptides for influenza A/M, CMV, or EBV [22]. These are common viral infections in humans, thus many individuals already have preexisting CD8^+^ T cells specific for these common viral infections. Furthermore, many of the viral antigenic peptide-specific CD8^+^ T cell epitopes have been reported [22]. These virus-specific CTL epitopes potentially can be used in conjunction with our approach.

## 5. Conclusions

Taken together, the chimeric PDL1-scFv-FC-RE7 protein concentrates and targets the antigen to the tumor as a result of PDL1 specificity and permits the cleavage and release of the CTL epitope such that the tumor becomes coated in the antigenic peptide and can be targeted by preexisting antigen-specific T cells to effectively kill the tumor cells. The chimeric protein technology can be used in conjunction with vaccines that can create preexisting antigen-specific CD8^+^ T cells to enhance antitumor effects. In this preclinical study, we use an HPV16 E7 antigenic system and CRTE7 DNA vaccine. Our strategy can be extended to other antigenic systems such as influenza, EBV, or CMV. Our approach serves as a potential treatment to circumvent the issues of immune tolerance and potentially can be applicable to many different tumors in the body.

## Data Availability

All data and materials are available from the corresponding author upon written request.
